# Editorial for the Special Issue “Applications of Biomedical Imaging and Sensing Technologies in Disease Diagnosis”

**DOI:** 10.3390/s26175668

**Published:** 2026-09-07

**Authors:** Marco Marcon

**Affiliations:** Department of Electronics, Information and Bioengineering, Politecnico di Milano, 20133 Milan, Italy; marco.marcon@polimi.it

## 1. Introduction

Biomedical imaging and sensing technologies are continuously reshaping disease diagnosis, precisely by linking measurement physics, signal processing, computer vision, artificial intelligence (AI), and clinical decision support. In the past, the main and most frequently encountered challenge was in acquiring diagnostically useful information: higher spatial resolution, better contrast, cleaner signals, and more stable measurements. Today, the challenge spans a broader scope. Diagnostic technologies must now also be affordable, repeatable, interpretable, deployable in real clinical environments, and compatible with the constraints of patients, clinicians, hospitals, and embedded platforms.

The scope of this Special Issue was therefore intentionally wide. It solicited contributions on magnetic resonance imaging (MRI), computed tomography (CT), ultrasound, positron emission tomography (PET), optical imaging, novel sensors, AI algorithms, and data-driven diagnostic methods. The papers accepted in this Special Issue confirm that the field is no longer limited to classical medical imaging. Instead, biomedical diagnosis increasingly hinges upon the interaction between sensors, computational models, acquisition protocols, and clinical workflows.

The eleven papers compiled in this Special Issue collectively provide a representative view of this transition. Some contributions focus on acquisition systems and sensor design; some address computational analysis, machine learning, or harmonization; others aim to reduce cost and increase accessibility. Taken together, these contributions show that progress in disease diagnosis depends on both technological innovation and translational awareness.

## 2. Biomedical Imaging and Sensing in the Current State of the Art

The broader state of the art shows that imaging- and sensing-based diagnosis is transitioning from proof-of-concept algorithms to clinical pathways. In ophthalmology, deep learning systems applied in optical coherence tomography and fundus imaging have demonstrated the potential to support referral decisions and autonomous screening in diabetic retinopathy [1,2]. In radiology, AI systems for head CT, lung cancer screening, and mammography have shown that large-scale datasets and reader studies can move algorithmic performance closer to that required for clinical utility [3,4,5]. Digital pathology has similarly benefited from weakly supervised approaches that are able to exploit whole-slide images at scale without requiring exhaustive pixel-wise annotations [6].

Simultaneously, the diagnostic landscape is expanding beyond conventional images. Multimodal dermatology combines visual information with clinical contexts [7]; breathomics and gas sensing explore metabolic signatures of disease [8,9]; ECG-based AI aims to extract structural cardiac information from inexpensive and widely available signals [10]; and retinal foundation models suggest that the eye may act as a window into systemic disease [11]. Population-level evaluation is also becoming increasingly important, as illustrated by AI-supported mammography trials that examine not only accuracy but also workload, interval cancers, and screening-program outcomes [12].

This evolution is relevant to the present Special Issue because it places the accepted papers in a clear context. The field is no longer driven exclusively by the question “Can the sensor or the algorithm detect the disease?”. A more pressing question is now emerging: “Can the complete system improve diagnosis in a reliable, efficient, and clinically meaningful way?”.

## 3. Contributions Published in This Special Issue

The papers published in this Special Issue can be grouped into five complementary themes: accessible and wearable sensing, optical and microscopy imaging, AI-supported image interpretation, MRI-based biomarkers, and transferable anomaly-detection methods. Throughout this section, the numbers in parentheses refer to the individual articles included in the Special Issue, as listed in the “List of Contributions” section.

### 3.1. Accessible Motion and Wearable Sensing

Bernal, Feipel, and Plaza developed and validated a Kinect-based motion capture system for gait analysis in individuals with anterolateral shoulder pain syndrome (Contribution 1). This work is relevant because it addresses a recurring barrier in clinical biomechanics: conventional motion-capture systems are accurate but costly, complex, and at times unsuitable in routine or low-resource settings. By comparing the proposed system with a commercial motion-capture reference, the authors showed that low-cost sensing can provide clinically useful kinematic information while reducing implementation complexity.

Pezzoli and colleagues proposed an eyewear-integrated infrared eye-tracking system based on photosensor oculography (Contribution 11). The study combines optical design, near-infrared emitters and photodiodes, modulation and demodulation, and lightweight regression models. Although evaluated in a controlled artificial-eye platform, the work is important for future smart eyewear, assistive technologies, and human–machine interfaces, where camera-free or low-power eye tracking may support accessibility and neurological or behavioral monitoring.

### 3.2. Optical Imaging, Skin Analysis, and Digital Microscopy

Spigulis and co-authors presented a triple spectral line imaging device for whole-body human skin assessment (Contribution 3). Their prototype combines a high-resolution camera with RGB laser spectral lines at 450, 520, and 628 nm, enabling multispectral skin imaging with narrow spectral bands. The study contributes to optical diagnostics by exploring a practical setup for whole-body skin evaluation and by reporting initial clinical data from volunteers.

Mori and colleagues introduced RT-4M, a real-time mosaicing manager for manual microscopy systems (Contribution 8). This contribution is particularly significant for digital pathology and bio-research facilities with limited budgets. Instead of requiring dedicated slide scanners or automated microscopes, the proposed system creates virtual slides from manual microscopy images and provides real-time preview during acquisition. The reported reduction in misalignment and the ability to work with fluorescent and hematoxylin-and-eosin-stained samples make the approach relevant for laboratories seeking a low-cost path toward digital workflows.

### 3.3. AI-Supported Medical Image Interpretation

Yu and colleagues proposed LPC-SonoNet, a lightweight network for fetal ultrasound standard plane detection (Contribution 4). Fetal ultrasound standard planes are essential for obtaining reproducible biometric measurements and for making prenatal diagnoses. The proposed light pyramid convolution strategy reduced the number of parameters compared with that seen with SonoNet64 while preserving, and slightly improving, classification accuracy. This is an example of an important trend in medical AI: performance must be balanced with computational efficiency if models are to be integrated into real-time imaging devices.

Li and co-authors developed LSDSL, a semi-supervised learning framework for medical image segmentation based on low-quality sensor data (Contribution 5). The work tackles a central issue in medical imaging: large labeled datasets are costly and often unavailable. By using hard-region exploration and pseudo-label sharing, the method improves the exploitation of labeled and unlabeled data across CT and MRI segmentation tasks. This contribution fits well within the increasing need for robust AI methods that can learn from imperfect, heterogeneous, and partially labeled clinical datasets.

Lee and colleagues proposed the AGS network for skin cancer classification (Contribution 7). The framework integrates augmentation, generative adversarial networks, and segmentation to address limited data availability and to improve lesion-focused classification. Tested with multiple deep learning classifiers on the HAM10000 dataset, the approach improved performance across architectures and achieved strong classification results. This paper highlights the continuing importance of data preparation, synthetic data generation, and lesion localization in dermatological AI.

### 3.4. MRI-Based Biomarkers, Quantitative Imaging, and Harmonization

Chudzik investigated whether structural information from standard T1-weighted MRI can support the machine-learning recognition of Parkinson’s disease stages (Contribution 6). Using volumes and spatial relationships of selected subcortical structures, this study compared several machine learning models and reported strong performance for differentiating healthy, prodromal, and Parkinson’s disease groups. This work illustrates the role of interpretable MRI-derived features in conducting neurodegenerative disease assessment.

Park and colleagues explored the prediction of germline BRCA mutations in high-risk breast cancer patients using machine learning and multiparametric breast MRI features (Contribution 9). This study combined morphologic, kinetic, and diffusion-related MRI biomarkers and revealed that features such as washout, background parenchymal enhancement, and tumor size were associated with mutation status. Although genetic testing remains the reference standard, this study suggests that MRI-derived biomarkers may support non-invasive risk stratification and personalized treatment planning.

Malyarenko and co-authors addressed the cross-scanner harmonization of AI/DL-accelerated quantitative bi-parametric prostate MRI (Contribution 10). This contribution is highly relevant because quantitative MRI is sensitive to acquisition protocols, reconstruction methods, scanner vendors, and bias in apparent diffusion coefficient and T2 mapping. By combining patient imaging and phantom-based validation, the authors proposed a workflow for harmonizing AI/DL-accelerated quantitative MRI across vendors, a key requirement for reproducible prostate imaging biomarkers.

### 3.5. Transferable Disease-Diagnosis Methods Beyond Human Medicine

Si and Kim presented CRASA, a generative adversarial serial autoencoder for chili pepper disease diagnosis using image reconstruction and background removal (Contribution 2). Although this paper is outside the scope of strictly human biomedical diagnosis, it is methodologically relevant to the Special Issue because it addresses image-based disease diagnosis through anomaly detection, reconstruction error, segmentation, and generative learning. Such methods are transferable to biomedical settings in which abnormality detection must be performed with a limited number of disease examples.

## 4. Cross-Cutting Themes

Several common themes emerge from these contributions.

Firstly, accessibility is a major driver of innovation. Kinect-based gait analysis, manual-microscopy mosaicing, lightweight fetal ultrasound classification, and smart-eyewear eye tracking all aim to reduce barriers to deployment. In diagnostic technologies, lower cost and reduced complexity are not secondary advantages; they can determine whether a method can be adopted outside highly specialized centers.

Secondly, the Special Issue highlights the increasing importance of AI under real-world data constraints. Medical datasets are often small, imbalanced, noisy, or weakly annotated. All of the papers on fetal ultrasound, semi-supervised segmentation, skin cancer classification, Parkinson’s disease MRI, and BRCA mutation prediction address different forms of this problem. The common takeaway between them is that model design must account for limited labels, heterogeneous inputs, and the need for computational efficiency.

Third, quantitative reliability and harmonization are becoming as important as raw performance. This is particularly evident in prostate MRI, where quantitative maps must remain comparable across scanners and protocols, but it is also relevant to optical imaging, microscopy, and wearable sensing. A diagnostic sensor is useful only if its output remains meaningful across devices, operators, patients, and time.

Fourth, the boundary between imaging and sensing is becoming increasingly porous. Eye tracking, gait capture, optical skin imaging, MRI, microscopy, ultrasound, and anomaly detection share a common diagnostic logic: the extraction of clinically meaningful information from structured measurements. This convergence is one of the most important directions for future advanced sensors.

## 5. Remaining Challenges and Future Directions

Despite the promising results presented, several challenges persist. The first challenge is external validation. Many diagnostic systems perform well in controlled or retrospective conditions but require broader evaluation across institutions, devices, populations, and acquisition protocols. The second challenge is interpretability. Clinicians need not only predictions, but also understandable evidence, uncertainty estimates, and quality-control indicators. The third challenge is workflow integration. A model that improves an isolated metric may fail to improve care if it increases workload, generates poorly calibrated alerts, or does not fit the clinical pathway.

Future work should therefore prioritize prospective validation, multi-center datasets, regulation-ready quality controls, and human-in-the-loop evaluation. For embedded and wearable systems, power consumption, calibration burden, anatomical variability, and usability must be considered from the beginning. For AI-based imaging systems, robustness to distribution shift, fairness across patient groups, and transparent reporting of failure cases will be essential.

## 6. Conclusions

This Special Issue demonstrates the breadth and vitality of current research in biomedical imaging and sensing technologies for disease diagnosis. The papers accepted and included in this Special Issue span the fields of hardware, software, machine learning, quantitative imaging, optical sensing, digital pathology, and wearable systems. Considered alongside the broader state of the art, these papers show that diagnostic innovation is evolving toward integrated systems that are accurate, efficient, accessible, interpretable, and clinically deployable. The next stage of advancements in the field will depend less on isolated algorithmic performance and more on the capacity to build validated diagnostic pathways in which sensors, imaging devices, AI models, and clinical expertise reinforce one another.
